# Upsurge in publications on ramp lesions of the meniscus: A bibliometric study

**DOI:** 10.1186/s43019-023-00190-6

**Published:** 2023-05-27

**Authors:** Riccardo D’Ambrosi, Srinivas B. S. Kambhampati

**Affiliations:** 1 IRCCS Istituto Ortopedico Galeazzi, Milan, Italy; 2 Dipartimento di Scienze Biomediche per la Salute, Università degli Studi di Milano, Milan, Italy; 3 Sri Dhaatri Orthopaedic, Maternity & Gynaecology Center, SKDGOC, Vijayawada, Andhra Pradesh 520008 India

**Keywords:** Ramp, Meniscal ramp, Bibliometrics, Arthroscopy

## Abstract

**Purpose:**

The purpose of this study was to analyze the trends in publications on ramp lesions of the meniscus in the current literature. We hypothesized that publications on ramp lesions have increased rapidly in recent years due to increased knowledge of both clinical and radiological pathology.

**Methods:**

A Scopus search performed on 21/01/23 retrieved 171 documents. A similar search strategy was employed to carry out a search for ramp lesions on PubMed with no time filters and only English articles. The articles were downloaded into Excel software, and citations for PubMed articles were determined from the iCite website. Analysis was performed using Excel. Using Orange software, data mining was performed from the titles of all articles.

**Results:**

There are a total of 126 publications from 2011 to 2022 with a total of 1778 citations in PubMed. Of all publications, 72% were published in the last 3 years, from 2020 to 2022, indicating an exponential increase in interest in this subject in recent years. Similarly, 62% of the citations were aggregated by the years 2017–2020, both years included. When the journals were analyzed according to the number of citations, the American Journal of Sports Medicine (AJSM) topped with 822 citations (46% of all citations) and 25 publications, followed by Knee Surgery, Sports Traumatology, Arthroscopy (KSSTA) with 27 articles and 388 citations (22% of all citations). When analyzed by citations per publication for different types of studies, randomized clinical trials (RCTs) were the most cited, with 32 citations per publication, followed by basic science articles with 31.5. Most of the basic science articles were cadaver studies examining anatomy, technique, and biomechanics. Technical notes were the third most cited at 18.64 per publication. While the USA is the country that leads publications, France is in a significant second position contributing to research on this topic, followed by Germany and Luxembourg.

**Conclusions:**

Global trend analysis suggests that ramp lesion research has significantly increased and that the number of papers on the topic is steadily increasing. We found that the publications and citations presented a rising trend, the majority of the highly cited papers were contributed by a few centers, and the most cited were randomized clinical trials and basic science studies. The long-term outcomes of conservatively and surgically treated ramp lesions have attracted the most research interest.

## Introduction

The posterior horn of the medial meniscus has received more attention over the past 10 years. The identification of lesions in the meniscosynovial zone, often referred to as ramp lesions, has opened up new approaches for the treatment of anterior cruciate ligament (ACL) injuries, with which they are frequently related [1]. Although Hamberg and Gillquist first identified these lesions in 1983 as “a peripheral vertical rupture in the posterior horn of the medial or lateral meniscus with an intact body,” interest in diagnosing and treating these lesions has only recently risen [2]. In a poll of directors of orthopedic sports medicine fellowship training programs in the USA, 61% of respondents stated that they first became aware of meniscal ramp lesions less than 7 years ago [3]. Ramp lesions were previously frequently underdiagnosed because they are challenging to diagnose using magnetic resonance imaging (MRI) or through the typical anterolateral portal view during arthroscopy [4, 5]. The posteromedial meniscocapsular junction is frequently inspected by up to 86% of the directors of orthopedic sports medicine fellowship training programs to detect ramp abnormalities [2]. In 2010, it was projected that these previously unidentified lesions affected 9% of the population [1, 2]. A prevalence between 20% and 30% was discovered in more recent investigations [4]. Ramp lesions are thought to account for approximately 50% of medial meniscus tears in ACL injury patients [6]. Untreated lesions may affect the medial meniscus’s integrity and, thus, the success of ACL restoration [6]. However, we are as yet unsure of the magnitude of the issue.

Bibliometrics is a research method used to provide the characteristics and development of a subject area [7]. It provides a useful way to assess the scientific effect of papers. The entities (such as nations, institutions, and writers) that have made the greatest contributions to science are identified in this type of analysis. The number of citations often indicates how interested academics are in utilizing journal articles in their research [7]. Consequently, bibliometric analysis can pinpoint research hotspots and anticipated trends in a specific field of study. No previous bibliographic analysis of the ramp lesion research has been found, according to an examination of the current literature. The purpose of this study was to analyze the trends of publications on ramp lesions of the meniscus in the current literature. We hypothesized that publications on ramp have quickly increased in recent years due to increased knowledge of both clinical and radiological pathology.

## Methods

A Scopus search was performed on 21/01/23 using the strategy (TITLE-ABS-KEY (meniscus) AND TITLE-ABS-KEY (ramp)), which retrieved 171 documents. A similar search strategy was employed to search PubMed for ramp lesions (("ramp lesion"[MeSH] OR "ramp meniscus"[MeSH] OR “ramp injury” OR “medial meniscus detachment”) with no time filters and only English articles. We retrieved 126 articles from PubMed.

### A flow chart of the article selection process is presented in Fig. 1 [8]

**Fig. 1:**
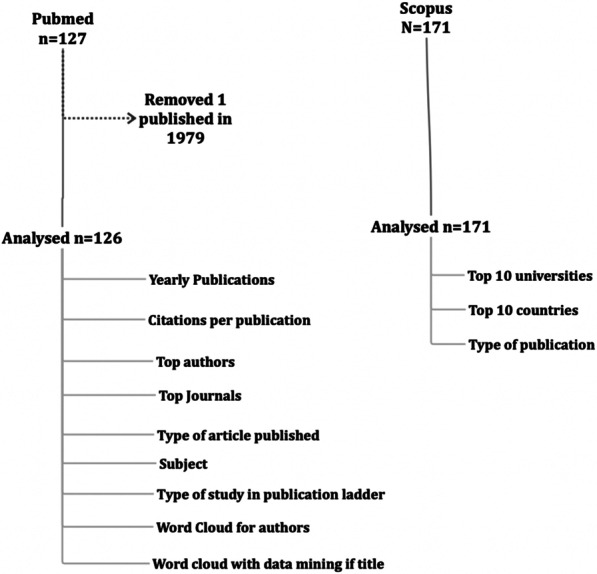
PRISMA type chart of analysis of articles and summary of methodology

The articles were downloaded into Excel software (MS Office 365), and citations for PubMed articles were determined from the iCite website (https://icite.od.nih.gov/) for PubMed. Analysis was performed using Excel. Data mining from the titles of all articles was performed using Orange software, Mac version 3.32.0 (https://orangedatamining.com/). The output is presented as word clouds. Most of the analysis was done using output from PubMed (yearly publications, citations per publication, top authors, and top journals). PubMed data were also analyzed regarding the type of article published. We created three additional columns in the PubMed database. Each column was created for a different type of article based on chronology (prospective/retrospective/cross sectional study), subjects (clinical, basic science, experimental, biomechanics), and type of study on the publication ladder [case report/case series or cohort study/randomized clinical trial (RCT)/systematic review/meta-analysis]. Scopus data were used to obtain data on the top 10 universities, countries, and types of publications only.

## Results

There are a total of 127 publications from 2011 to 2022 with a total of 1778 citations in PubMed.

One publication from 1979 was picked up by PubMed in this search strategy. It turned out that this publication mentioned the word “ramp” in the abstract but is not related to meniscus and hence was removed from the analysis, leaving 126 manuscripts.

**Fig. 2:**
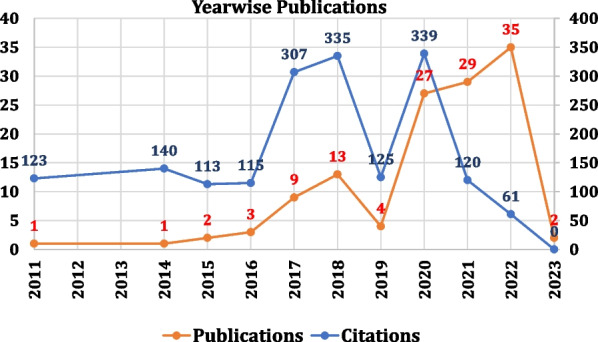
Yearwise publications and citations on ramp lesions

Of all publications, 72% were published in the last 3 years, from 2020 to 2022 (Fig. 2), indicating exponential interest in this subject in recent years. While the number of publications was only 4 in 2019, they jumped to 27, an almost eight-fold increase, in 2020. Similarly, 62% of the citations were aggregated by the years 2017–2020, both years included. The increase in both the number of publications and citations in recent years indicates the amount of interest this topic has gained in recent years.

**Fig. 3:**
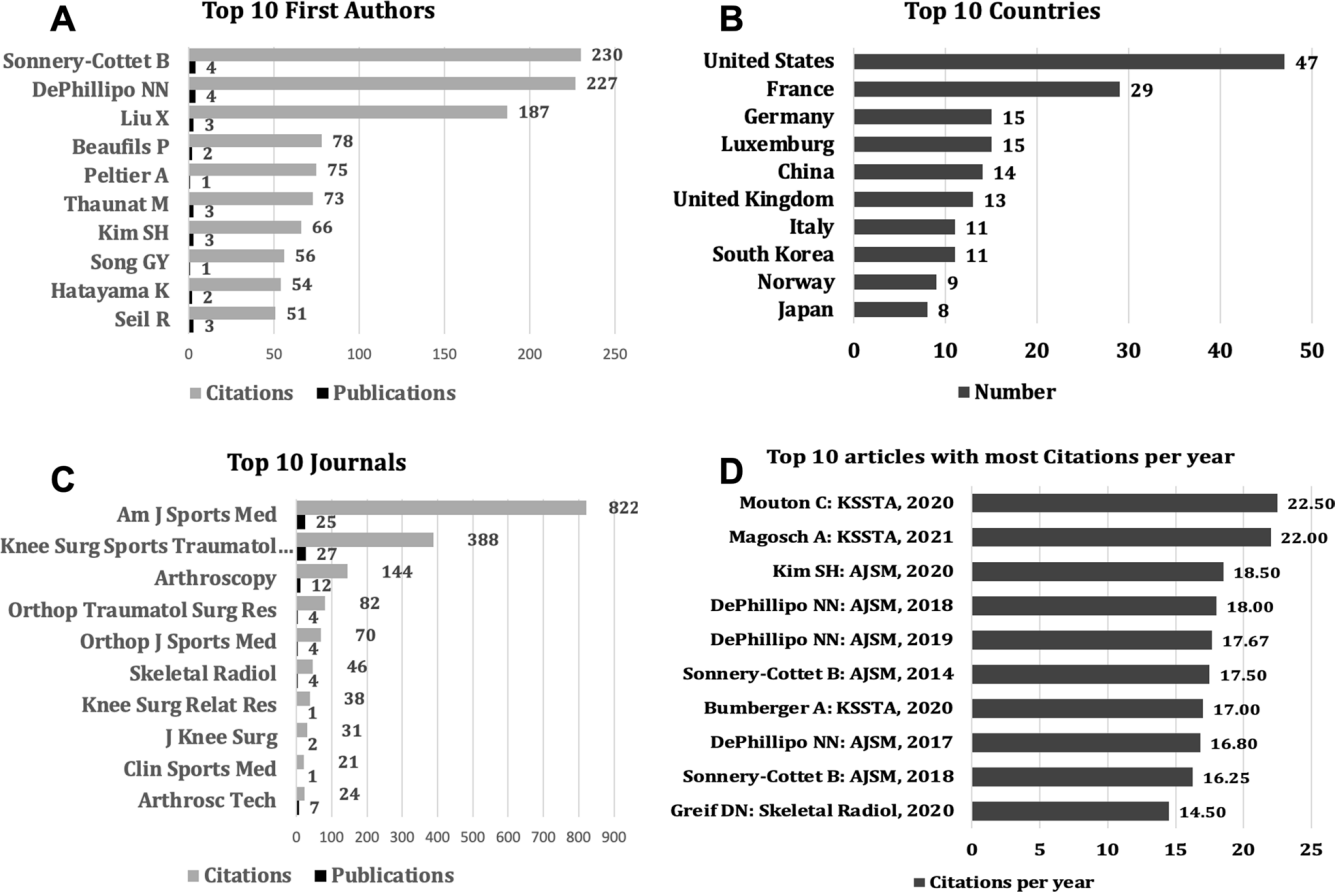
**A** Top 10 first authors; **B** top 10 countries (Scopus); **C** top 10 journals; and **D** top 10 articles with names of authors and journals arranged according to citations per year

When we arranged authors according to the most cited (Fig. 3A), Sonnery-Cottett topped the list with 230 citations and four publications, followed by DePhillipo with 227 citations and four publications.

**Fig. 4:**
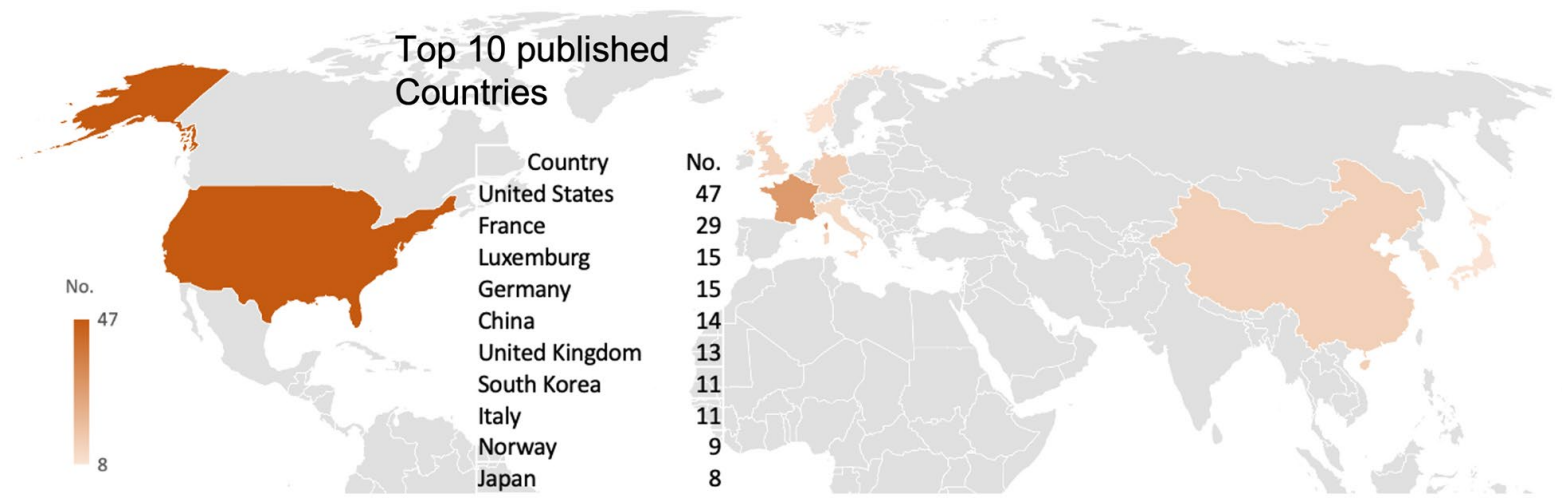
World map of worldwide research productivity for ramp lesions

We looked into the Scopus data to examine the top 10 countries and types of articles, as this information is not given in the PubMed output. While the USA is the country that leads publications (Figs. 3B and 4), France is in a significant second position contributing to research on this topic, followed by Germany and Luxembourg. Analyzing by continents, Europe has 92 publications, North America reports 47 articles, and Asia 34.

When the journals were analyzed according to the number of citations (Fig. 3C), the American Journal of Sports Medicine (AJSM) topped with 822 citations (46% of all citations) and 25 publications, followed by Knee Surgery, Sports Traumatology, Arthroscopy (KSSTA) with 27 articles and 388 citations (22% of all citations). AJSM and KSSTA contributed 52 (41% of all publications). The top three indexed journals on arthroscopy—AJSM, KSSTA, and Arthroscopy—accounted for 76% (1354) of all citations on ramp lesions.

At least two studies mentioned peripheral posterior horn tears rather than using the term “ramp lesions” in the literature before the first publication in 2011 [9, 10]. When we assessed the first authors according to the citations per year for each publication, DePhillipo NN appeared three times and was involved in three of the top 10 articles arranged according to citations per year [11–13]. The maximum number of citations per year was 22.5 by Mouton [14] (Fig. 3D). Nine of the top 10 cited articles per year were published in the last 5 years. The most cited per year was published in 2020 [15].

**Fig. 5:**
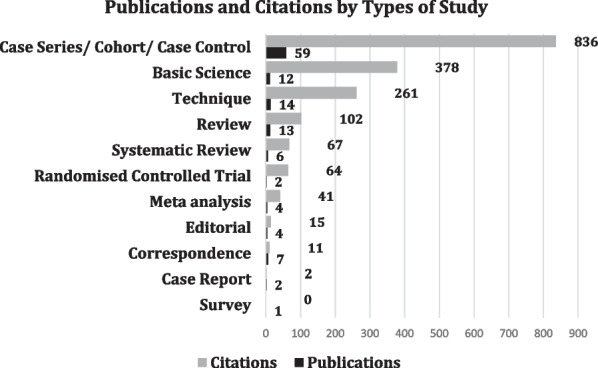
Publications and citations based on type of study

We classified all the articles based on type of study (Fig. 5) and examined the number of publications and citations according to the type of study. The fewest articles were survey and case reports, amounting to just three articles. Clinical studies (case series, cohort studies, and case control studies) were grouped into one category and were found to be the most published and cited, with 59 and 836 studies, respectively. The majority of these were cohort studies. This was followed by 12 basic science articles with 378 citations. There were 10 systematic reviews/meta-analyses with 108 combined citations and only two RCTs with 64 citations.

When analyzed by citations per publication for different types of studies, RCTs were the most cited, with 32 citations per publication, followed by basic science articles with 31.5. Most of the basic science articles were cadaver studies examining anatomy, technique, and biomechanics. Technical notes were the third most cited, at 18.64 per publication.

**Fig. 6:**
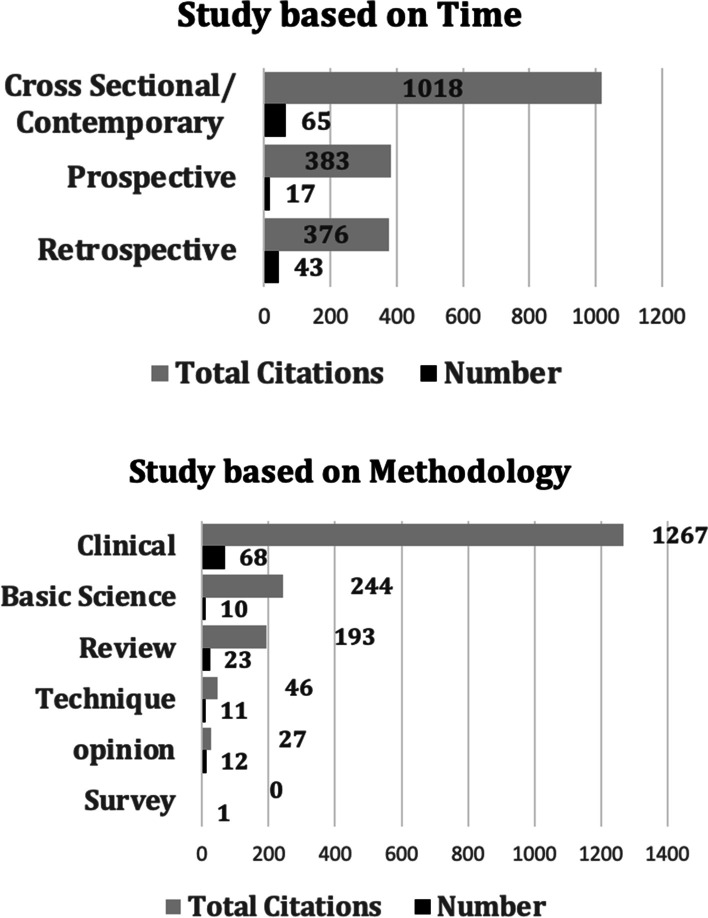
**A** Top cited studies based on time; **B** top cited studies based on methodology

**Fig. 7:**
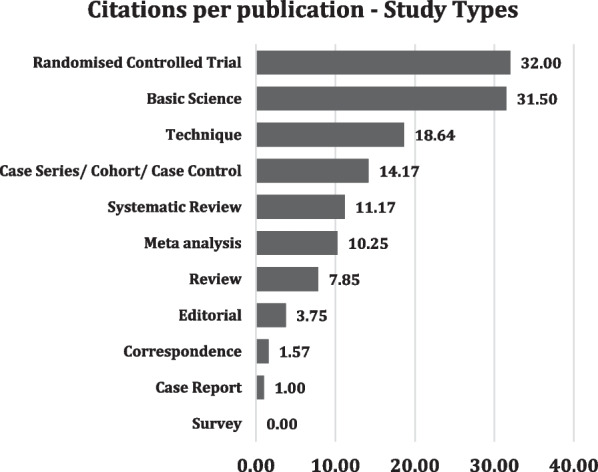
Top cited studies in the evidence ladder

When we classified articles based on time, methodology (Fig. 6A, B), and level of evidence types (Fig. 7), we excluded one study since it was a neurology article and not related to ramp lesions. The results of classifying the studies are given in the charts. When classified by time, cross-sectional or contemporary studies were the most frequent and cited, with 65 and 1018 citations, respectively. When based on methodology, clinical studies were the most frequent and cited, with 68 and 1267 studies, respectively. When classified according to studies in the evidence ladder, case series/cohort and case control studies were the most frequent, with 59 and 836 studies, respectively. However, when we looked at the citations per publication, RCTs were the most cited per paper, with 32, closely followed by basic science articles at 31.5, followed by techniques with 18.6 at a distant third.

Regarding the type of publications in Scopus, articles are the most common type, with 127, followed by reviews, with 28, and editorials, with 6. Conference papers (5), notes (2), book chapters (1), erratum (1), and letters (1) were very few in number.

**Fig. 8:**
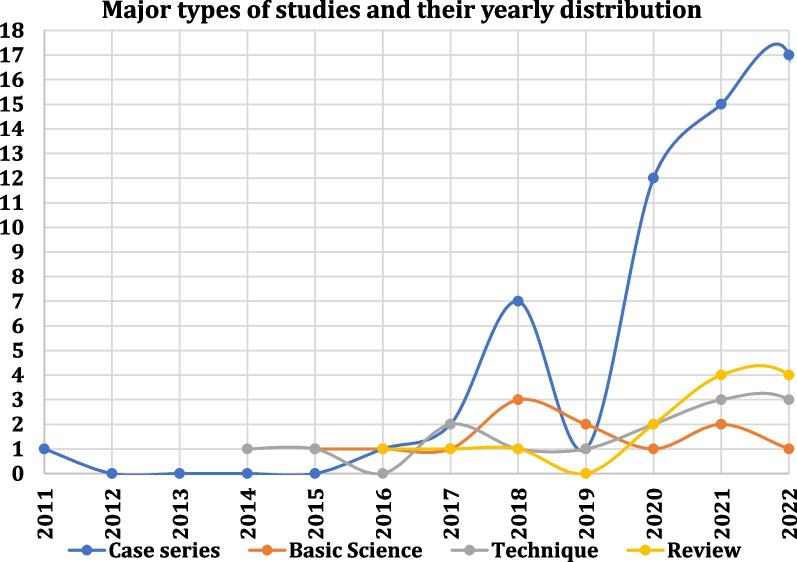
Yearly distribution of types of studies on ramp lesions

Tracing of yearly publications (Fig. 8) showed a prominent graph for case series in recent years starting in 2019. The graphs of studies on basic science, technique, and review appear similar.

**Fig. 9:**
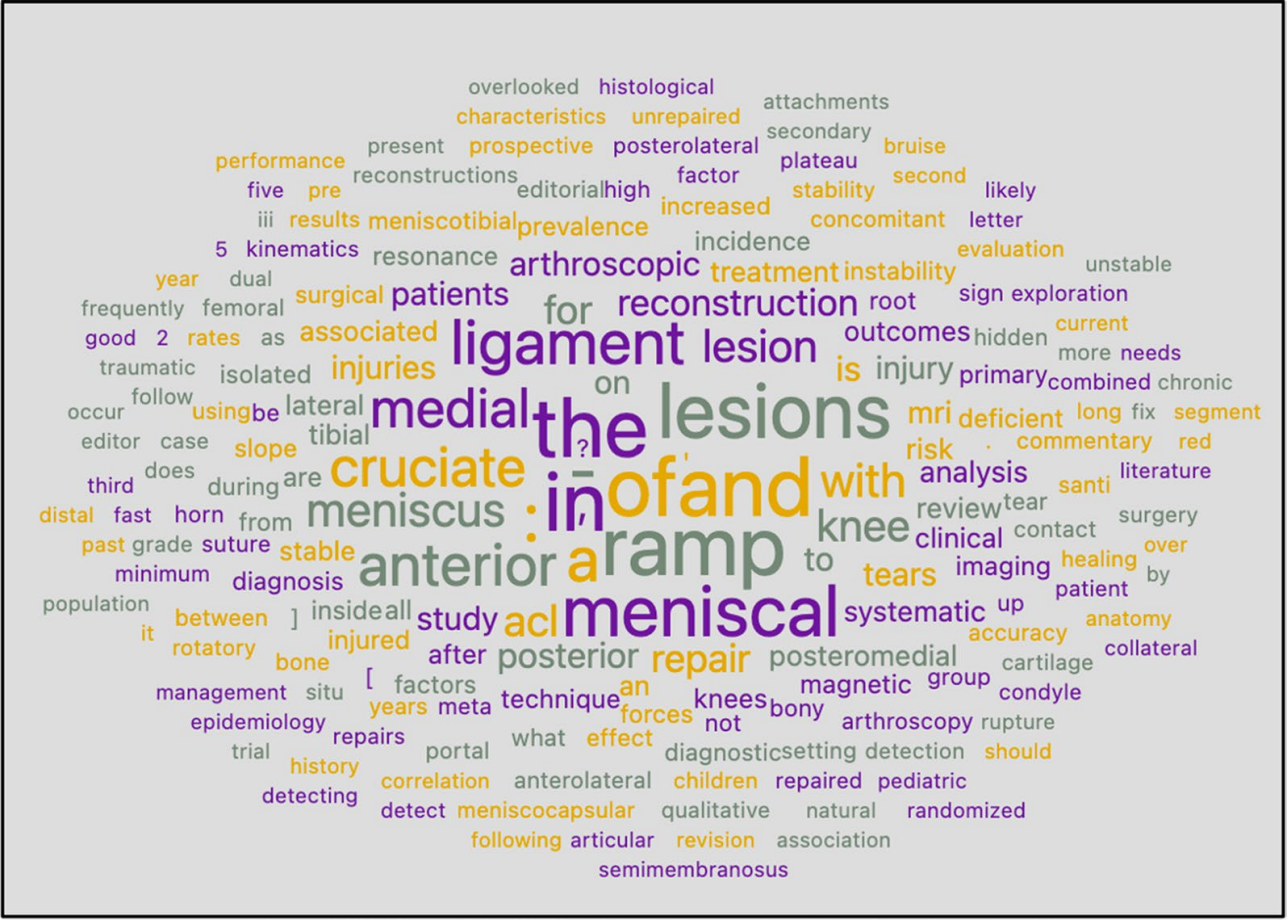
Word cloud of titles of articles from PubMed

A word cloud was derived from using Orange software on the titles of all articles (Fig. 9). The most prominent words other than “ramp” and “meniscus” include “anterior,” “cruciate,” and “medial,” indicating medial meniscus and association with ACL injury of this lesion. “Repair,” “reconstruction,” and “posterior” are other common words indicating management and portal/location of the lesion.

**Fig. 10:**
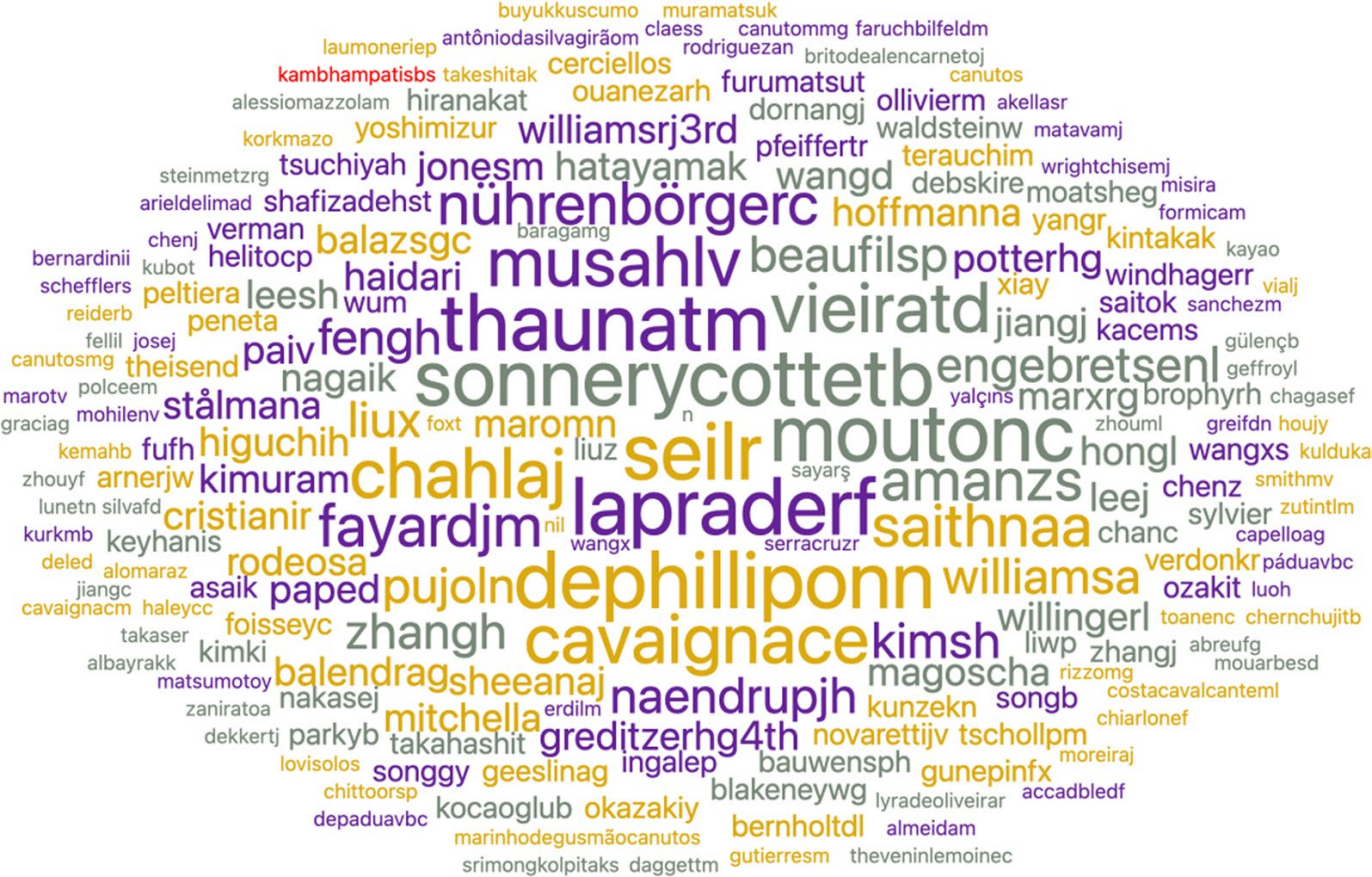
Word cloud of all authors in any position from PubMed

The word cloud from authors of all articles (Fig. 10) showed the most prominent names of authors in the word cloud to be Seil, Lee, Kim, LaPrade, Sonnery-Cottet, Wang, DePhillippo, Thaunat, and Mouton. Lee, Kim, and Wang are common surnames and possibly belong to different authors, although a single author could be a possibility. The top five published authors in any place in the article belong to this group of names.

## Discussion

The most important finding of the current study confirms our hypothesis, showing that almost all literature (> 70%) regarding ramp lesions has been published in the last 3 years. The interest in this topic is also demonstrated by the fact that clinical studies were found to be the most published and cited articles.

“Ramp lesion” is a relatively new term in the literature, and although it was first described in 1988, significant publications only started 12 years ago in 2011. Although posterior meniscus injury has been described in the literature before, the concept of ramp lesion is a relatively new one, with Strobel describing it in 1988 and the injury classified only recently [2]. Despite the long history of ramp lesion detection, the subject has received little research attention over the past few decades until a recent uptick in interest [2, 6]. This underestimation of their incidence due to a high rate of missed diagnoses, lack of understanding of their biomechanical effects, and an intuitive belief that these lesions may heal on their own are probably the causes of the topic’s lack of prior relevance [16, 17]. The increased attention given to these injuries in recent years signals both a growing understanding of their significance and the link between them and posteromedial knee instability. According to recent literature [15–17], the posterior meniscocapsular junction and/or the posterior meniscotibial ligament are torn in these injuries, The phrase “hidden lesion,” which refers to the challenge of recognizing ramp lesions via standard anterior arthroscopic portals as well as with preoperative MRI, which has low sensitivity, has also recently been used to describe this injury [15–17]. As reported by Sonnery-Cottet et al., it is interesting that all over the world, in orthopedic meetings, the meniscal root lesion is more extensively debated than the ramp lesion. This is despite the fact that ramp lesions are a lot more common [24% in ACL reconstruction (ACLR) in our experience]. This greater focus on root tears is perhaps because of the well-recognized loss of hoop force distribution and profound biomechanical consequences of root tears. However, it is increasingly recognized that large ramp lesions also have important biomechanical consequences, including persistent anteroposterior and rotational laxity and an association with higher ACL graft failure rates [2].

In 2016, Thaunat et al. proposed a classification of the medial meniscosynovial or meniscocapsular tears into five types: Type 1 refers to meniscocapsular lesions that are located very peripherally in the synovial sheath. Mobility at probing is very low. Type 2 refers to partial superior lesions that are stable and can only be diagnosed through a trans-notch approach. Mobility at probing is low. Type 3 refers to partial inferior or hidden lesions that are typically subtle or not immediately visible, even with trans-notch visualization. However, they can be strongly suggested by significant mobility on probing and identification of abnormal tissue quality on needling. Type 4 refers to a complete tear of the red-red zone with mobility at probing being very high. Type 5 refers to a double tear involving the meniscocapsular junction and a second, more anterior tear of the posterior horn [17].

Numerous potential processes are thought to contribute to ramp lesions [18, 19]. The strongest stresses are delivered through the posteromedial capsule during valgus strain, internal tibial rotation, and axial loading during ACL injuries [6]. This causes the most straightforward injury and provides a potential mechanism because it causes impaction between the medial femoral condyle and the medial aspect of the tibial plateau, trapping the meniscus. Compensatory varus alignment and internal rotation of the femur occur after the original pivot-shift process [15].

Based on a growing understanding of the possible significance of these injuries in terms of maintaining knee stability, the concept of ramp lesions as posteromedial instability is beginning to take shape. In ACL-deficient cadaveric knees, Ahn et al. and Peltier et al. have shown an increase in anteroposterior instability. Other studies have shown significant increases in internal and external rotation laxity at all knee flexion angles following ramp lesions to the meniscotibial ligament [20, 21].

Finochietto’s jump sign was described in 1935 for posterior lesions of the semilunar cartilages [22]. Although the word “ramp” was not used here, it is apparent that a similar lesion was described. This was later confirmed by other authors [23, 24]. Losee reported it as a pathognomonic sign of longitudinal tear of the posterior meniscus [23]. Espejo-Baena confirmed this sign as pathognomonic by clinical and arthroscopic examinations [24].

While cohort studies provide evidence of the regular variations within a condition, case reports provide the extreme variations and rarer aspects of the condition. There are only two case reports on ramps, with just two citations for one of them [25, 26]. However, both were recently reported, and hence, citations are expected to be low at this stage for any publication.

A survey among surgeons on the condition indicates an effort to gain insight into the understanding of the condition among the surgeons and to standardize understanding of the subject as well as management aspects of the condition. It must be noted that the main subject was ACL surgical trends, and ramp lesions were only part of the survey [27].

The American Journal of Sports Medicine has the highest number of citations (822) with the second highest number of publications (25), followed by Knee Surgery, Sports Traumatology, Arthroscopy (27 articles and 388 citations). As these journals were most favored by researchers around the world, their high reputations and authority regarding orthopedics research are implied.

It is also interesting to analyze the nations that published the highest number of publications, and the USA dominate by far (47), followed by France (29) and Germany (15). These results are totally in line with similar publications analyzing different knee pathologies, such as knee osteoarthritis, stem cell therapy for knee disease, and usage of unicompartmental knee arthroplasty (UKA), where the USA is always reported to be the country with the highest number of publications, followed by England and China [28–30]. However, when we look at the continents, Europe (92) has published more than North America (47).

This study has a number of restrictions. First, the study excluded non-English literature and solely searched the Scopus and PubMed databases, which could have introduced biases. Analysis of multiple databases cannot be done in such studies because of the differing methods of calculating citation numbers between the search engines. Another potential limitation of the study relates to the criteria for inclusion and exclusion of articles; in fact, we decided not to exclude surgical techniques and editorials from our analysis; these types of articles usually have reduced scientific value compared with clinical trials or meta-analyses. Bibliometric studies generally do not exclude any articles unless they are focused studies. As ours is not a focused study, and since literature on ramp lesions is in its infancy and evolving, we did not exclude any articles. Our study could serve as a baseline to compare with any future studies on this topic. Due to the short after-publication time, recently published high-quality documents have a low citation frequency, and the evaluation of the papers’ quality could be subjective. Bibliometrics only describes the broad trend in a particular topic. The diverse statistical algorithms utilized by various software packages might potentially cause errors. Despite the potential drawbacks stated above, we think the methodology used in this study is acceptable for the type of study. Research trends and hotspots can be visually exhibited through bibliometrical and visual analysis, which can be used to identify future paths for clinical and basic research and boost research proficiency in the ramp lesion field.

## Conclusions

Global trend analysis suggests that ramp lesion research has significantly increased and that the number of papers on the topic is increasing steadily. We found that the publications and citations presented a rising trend, the majority of the highly cited papers were contributed by a few centers, and the most cited were randomized clinical trials and basic science studies. The long-term outcomes of conservatively and surgically treated ramp lesions have attracted the most research interest.

## Data Availability

The datasets used during and/or analyzed during the current study are available from the corresponding author on reasonable request.
